# Health Care Use Preceding Suicide by Firearm Compared with Suicide by Other Means — Alaska, Colorado, and Washington, 2020–2022

**DOI:** 10.15585/mmwr.mm7421a2

**Published:** 2025-06-12

**Authors:** Julie E. Angerhofer, Maricela Cruz, Jennifer Shaw, Christine Stewart, Artie Runkle, Erika Wolter, Erika Holden, Shannon Medlock, LeeAnn Quintana, Elena Noon Kuo, Juanita Trejo, Roxanna King, Jennifer Boggs

**Affiliations:** ^1^Kaiser Permanente Washington Health Research Institute, Seattle, Washington; ^2^Department of Health Systems and Population Health, University of Washington School of Public Health, Seattle, Washington; ^3^University of Alaska Fairbanks, Center for Alaska Native Health Research, Fairbanks, Alaska; ^4^Kaiser Permanente Colorado Institute for Health Research, Aurora, Colorado; ^5^Southcentral Foundation Data Services, Anchorage, Alaska; ^6^Southcentral Foundation Research Department, Anchorage, Alaska.

**Summary**

**What is already known about this topic?**

Many persons at risk for suicide seek health care before their death by suicide, but patterns of care use might be different for persons who die by firearm suicide than for those who die by other suicide means.

**What is added by this report?**

Approximately one half (54.6%) of 683 suicide decedents whose deaths were recorded by three health care organizations in Alaska, Colorado, and Washington during 2020–2022 died by firearms. Among the decedents who received care from these organizations, use of mental health care services was significantly less in the year before death among persons who died by firearm suicide than among those who died by other suicide means.

**What are the implications for public health practice?**

Persons who die by firearm suicide might not access mental health care services. Suicide prevention practices in health care, designed to help identify and engage persons at risk in supportive care, need to reach beyond mental health encounters, particularly for firearm suicide prevention.

## Abstract

Firearms are the most common means of suicide in the United States and a leading cause of death among all persons aged 10–64 years. Most persons who die by suicide see a clinician in the year preceding their death; thus, health care encounters are important opportunities for suicide prevention. Persons who die by firearm suicide differ demographically and clinically from those who die by other suicide means, suggesting that opportunities for prevention might also differ between these groups. This report examined patterns of health care use in the year preceding suicide death to identify potential opportunities for prevention among persons who died by firearm suicide and those who died by other means of suicide. State cause-of-death records for 2020–2022 were linked to electronic health records from health systems in Alaska (Southcentral Foundation) and Colorado and Washington (both Kaiser Permanente). Quarterly past-year health care use preceding death was examined across service settings, including primary care, outpatient mental health specialty care, emergency care, and inpatient care. During 2020–2022, across the three health systems, 683 persons died by suicide. The majority of these deaths (54.6%) occurred by firearm. Patterns of past-year health care use preceding suicide were similar for persons who died by firearm and other suicide means, with the exception of mental health care, which was significantly lower in specialty and primary care settings. These findings suggest that many persons who die by firearm suicide do not access mental health care before their death. Suicide prevention practices in health care, designed to help identify and engage persons at risk in supportive care, need to reach beyond mental health encounters, particularly for firearm suicide prevention.

## Introduction

Suicide is the second leading cause of death in the United States among persons aged 10–44 years, and firearms are the most common means.* Health care encounters might represent important suicide prevention opportunities. Research has demonstrated that approximately 45% of persons who die by suicide have seen a clinician in the month before death, and most have seen a clinician in the year preceding suicide (*1*). Firearm suicide decedents have different demographic and clinical characteristics than do those who die by other means of suicide (*2*,*3*). For example, one recent study found that firearm suicide rates were highest among military service members, men, adults aged ≥65 years, and those living in rural areas (*2*). Another study found that persons who died by firearm suicide were less likely to have a diagnosed mental health condition, a substance use condition, or a previous suicide attempt documented in their medical record than did those who died by other suicide means (*3*).

Less clear is whether or how these demographic and clinical differences might affect prevention opportunities in health care systems. Research on care use preceding suicide death indicates that, whereas many decedents used primary health care services in the months preceding death, a smaller proportion used mental health specialty care (*1*). How these patterns in rates of care use might differ among those who die by firearm versus other suicide means is unknown.

This report examined patterns of health care use among persons who died by firearm suicide compared with those who died by other means of suicide in three large U.S. health care systems, to identify whether and how those patterns might differ among persons at risk for firearm suicide. Study results could help guide development and implementation of suicide prevention practices in health care systems nationwide.

## Methods

### Data Source and Study Population

State death records and electronic health records (EHRs) were obtained from three participating nonprofit health care systems, jointly serving more than one million persons. These included the Colorado and Washington regions of Kaiser Permanente, which are integrated insurance and health care providers each serving approximately 500,000 members, and the Southcentral Foundation, which serves approximately 70,000 American Indian and Alaska Native persons in the southcentral region of Alaska. Approvals to conduct these analyses were obtained from the Kaiser Permanente institutional review board, the Southcentral Foundation and Alaska Native Tribal Health Consortium research review committees, and the Indian Health Service Alaska Area institutional review board.

State medical examiner cause-of-death records were linked to EHR data to identify all persons enrolled in the three participating health care systems who died by suicide during 2020–2022. All Kaiser Permanente members and Southcentral Foundation customer-owners,^†^ collectively referred to as patients in this report, aged ≥13 years who received care at least once in the 3 years preceding death, were included in the study sample. This inclusion was consistent with population-based denominator definitions (e.g., “enrolled” or “empanelled”) used by care delivery leaders to determine how to improve the quality of care (i.e., prevention) and improve outcomes among patients considered to be actively receiving care from their organizations.

### Data Measures

Cause-of-death indicators for means of death were defined using *International Classification of Diseases, Tenth Revision, Clinical Modification* (ICD-10-CM) mortality codes for intentional self-harm (X60–X84 and Y87.0), further categorized as having occurred by firearm (X72–X74) or nonfirearm means.^§^ Care use measures included all past-year health care visits preceding death for each suicide decedent and were categorized into subgroups of use using the service setting type; EHR data were supplemented with claims data at Kaiser Permanente sites. These health care visits included virtual or in-person primary care, urgent care, emergency care, inpatient care, and mental health specialty care encounters between a health care provider and patient. Asynchronous encounters, such as EHR-based reminders and messages were not included. Mental health ICD-10-CM codes (as defined in the Mental Health Research Network diagnosis codes)^¶^ were linked with health care encounters to better distinguish health care encounters that included a mental health component from health care provided for other conditions. Self-reported firearm access and patient characteristics known to be associated with suicide were used to describe the study population, on the basis of previous research at Kaiser Permanente Washington (*4*). Variables extracted from EHR data included patients’ sociodemographic characteristics (age, sex, race and ethnicity, rural residency, and education [measured as percentage of the population with a college degree stratified by U.S. Census Bureau tract]), clinical characteristics (depression, anxiety, substance use disorders, suicide attempt, illness, and medical comorbidity [measured using the Charlson Comorbidity Index]) (*5*), and source of health insurance coverage.

### Statistical Analysis

Analyses compared demographic and clinical characteristics of persons who died by firearm suicide with those of persons who died by other suicide means. Statistically significant differences were identified using Pearson's chi-square tests. Care-use plots were generated to examine quarterly use of health services received in primary care, outpatient mental health specialty care, emergency care, and inpatient care service settings in the year preceding death. Asymptotic 95% CIs were constructed based on the normal distribution. The mean and median number of visits across these service settings, during the 3, 6, and 12 months preceding death, were also calculated among those who received any care during the same time. Two-sided t-tests (α = 0.05) were conducted to compare (among persons who used care) the mean number of health care visits among those who died by firearm suicide with the number among those who died by other suicide means. Post-hoc stratified analyses were added to further explore differences in care use patterns among males and females who died by firearm suicide versus other means.

## Results

### Sociodemographic and Clinical Characteristics

A total of 683 suicide deaths were recorded by three health care organizations in Alaska, Colorado, and Washington during 2020–2022, including 373 (54.6%) deaths by firearm and 310 (45.4%) by other means (Table). A higher proportion of males compared with females died by firearm suicide (87.9 versus 12.1%; p<0.01) and other means of suicide (62.6% versus 37.4%; p<0.01). The average age of persons who died by firearm suicide (50 years) was higher than those who died by other means (45 years; p<0.01).

**TABLE:** Characteristics of patients who died by firearm suicide versus other means of suicide — three health care systems,* Alaska, Colorado, and Washington, 2020–2022

Characteristic	No. (%)			p-value^†^
	All suicide decedents N = 683	Means of death		
		Firearm n = 373	Other n = 310	
**Age group, yrs**				
13–17	**27 (4.0)**	15 (4.0)	12 (3.9)	<0.01
18–39	**246 (36.0)**	127 (34.0)	119 (38.4)	
40–64	**248 (36.3)**	119 (31.9)	129 (41.6)	
≥65	**162 (23.7)**	112 (30.0)	50 (16.1)	
Mean (SD)	**48 (20)**	50 (22)	45 (19)	
**Sex**				
Female	**161 (23.6)**	45 (12.1)	116 (37.4)	<0.01
Male	**522 (76.4)**	328 (87.9)	194 (62.6)	
**Race and ethnicity^§^**				
American Indian or Alaska Native	**40 (5.9)**	13 (3.5)	27 (8.7)	<0.01
Asian or Pacific Islander	**18 (2.6)**	7 (1.9)	11 (3.5)	0.26
Black or African American	**18 (2.6)**	12 (3.2)	6 (1.9)	0.42
Hispanic or Latino	**44 (6.4)**	16 (4.3)	28 (9.0)	0.02
Native Hawaiian or Pacific Islander	**6 (0.9)**	4 (1.1)	2 (0.6)	—
White	**397 (58.1)**	236 (63.3)	161 (51.9)	<0.01
Other	**20 (2.9)**	12 (3.2)	8 (2.6)	0.79
Multiracial	**7 (1.0)**	6 (1.6)	1 (0.3)	—
Unknown	**166 (24.3)**	85 (22.8)	81 (26.1)	0.36
**Health care insurance**				
Commercial	**349 (51.1)**	188 (50.4)	161 (51.9)	<0.01
Indian Health Service	**32 (4.7)**	9 (2.4)	23 (7.4)	
Medicaid	**14 (2.0)**	6 (1.6)	8 (2.6)	
Medicare	**178 (26.1)**	119 (31.9)	59 (19.0)	
Private pay	**83 (12.2)**	39 (10.5)	44 (14.2)	
Other or none	**27 (4.0)**	12 (3.2)	15 (4.8)	
**Percentage of decedent’s U.S. Census Bureau tract population with college degree^¶^**				
<25%	**505 (73.9)**	279 (74.8)	226 (72.9)	0.62
≥25%	**143 (20.9)**	83 (22.3)	60 (19.4)	
**Percentage of decedent’s U.S. Census Bureau tract population earning >200% of FPL^¶^**				
<75%	**613 (89.8)**	345 (92.5)	268 (86.5)	0.47
≥75%	**35 (5.1)**	17 (4.6)	18 (5.8)	
Residence**				
Rural	**25 (3.7)**	11 (2.9)	14 (4.5)	0.42
Suburban	**248 (36.3)**	141 (37.8)	107 (34.5)	
Urban	**410 (60.0)**	221 (59.2)	189 (61.0)	
**Mental health diagnosis in year preceding death^††^**				
Anxiety	**214 (31.3)**	93 (24.9)	121 (39.0)	<0.01
Chronic serious mental illness	**63 (9.2)**	27 (7.2)	36 (11.6)	0.07
Depression	**206 (30.2)**	95 (25.5)	111 (35.8)	<0.01
Substance use problem	**110 (16.1)**	48 (12.9)	62 (20.0)	0.02
Suicidal self-harm or suicide attempt	**62(9.1)**	26 (7.0)	36 (11.6)	0.03
**Charlson Comorbidity Index score^§§^**				
0	**564 (82.6)**	292 (78.3)	272 (87.7)	<0.01
1	**39 (5.7)**	23 (6.2)	16 (5.2)	
≥2	**80 (11.7)**	58 (15.5)	22 (7.1)	

**Abbreviation:** FPL = federal poverty level.

* Southcentral Foundation (Alaska) and Kaiser Permanente (Colorado and Washington).

^†^ Calculated using Pearson's chi-squared tests; values not reported when sample sizes were not large enough to meet test conditions.

^§^ Categories sum to >100% of patient sample because all persons who identified as Hispanic or Latino are included in this category. All persons who identified as multiple races identified as multiracial. The other race and ethnicity group includes responses that are not possible to categorize into one of the broader categories (e.g., Irish, Ashkenazi Jewish, or human race).

^¶^ U.S. Census Bureau tract variables available for Kaiser Permanente patients only.

** On the basis of a condensed version of the 2013 National Center for Health Statistics urban-rural categorization for counties. Urban = large central metropolitan, large suburban = large fringe metropolitan (described as a large suburban area in data briefs), smaller suburban = medium metropolitan and small metropolitan, and most rural = micropolitan and noncore.

^††^ Mental health diagnostic codes used to create the analytic data set used for this analysis are available (2024 Diagnosis Codes – Mental Health Research Network); chronic serious mental illness diagnoses include bipolar disorder, schizophrenia, other psychosis, or personality disorders.

^§§^ Predicts the mortality for a person on the basis of 17 comorbid conditions.

Many sociodemographic characteristics were similar among persons who died by firearm suicide and those who died by other means of suicide. However, in the year preceding death, a lower percentage of persons who died by firearm suicide had received a mental health diagnosis than had those who died by other means of suicide, including anxiety (24.9% versus 39.0%; p<0.01), chronic serious mental illness (7.2% versus 11.6%; p = 0.07), depression (25.5% versus 35.8%; p<0.01), substance use problem (12.9% versus 20.0%; p = 0.02), and nonfatal suicidal self-harm or suicide attempt (7.0% versus 11.6%; p = 0.03). In contrast, a higher percentage of firearm suicide decedents had a Charlson Comorbidity Index score ≥2 (a medical comorbidity associated with increased risk for death) than did those who died by other suicide means (15.5% versus 7.1%; p<0.01).

### Health Care Use

**FIGURE 1:**
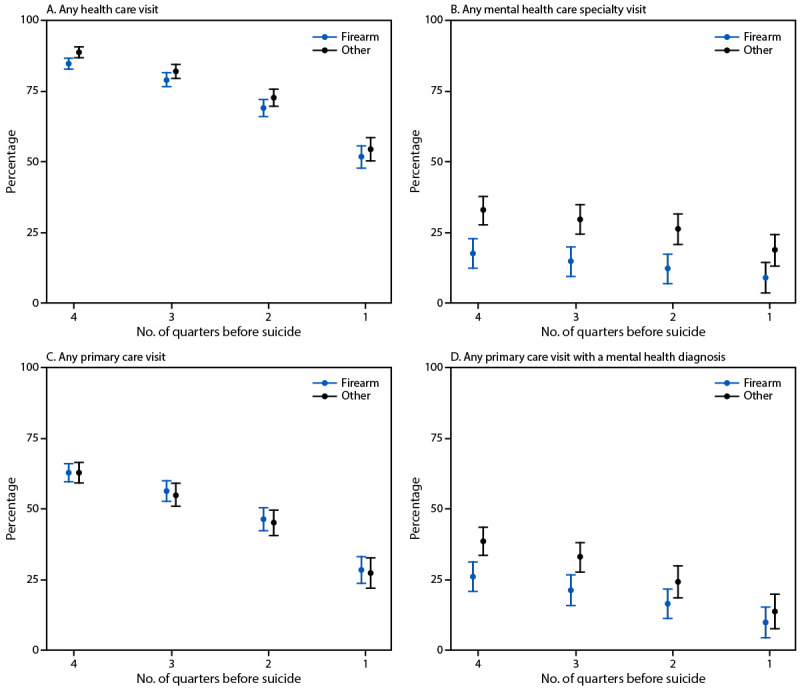
Percentage* of suicide decedents who had any health care visit (A), any mental health care specialty visit (B), any primary care visit (C), or any primary care visit with a mental health diagnosis (D), in the year preceding suicide, by means of suicide (firearm or other) — three health care systems,^†^ Alaska, Colorado, and Washington, 2020–2022 * Values are cumulative over the entire period; 95% CIs are indicated by bars. ^†^ Southcentral Foundation (Alaska) and Kaiser Permanente (Colorado and Washington).

**FIGURE 2:**
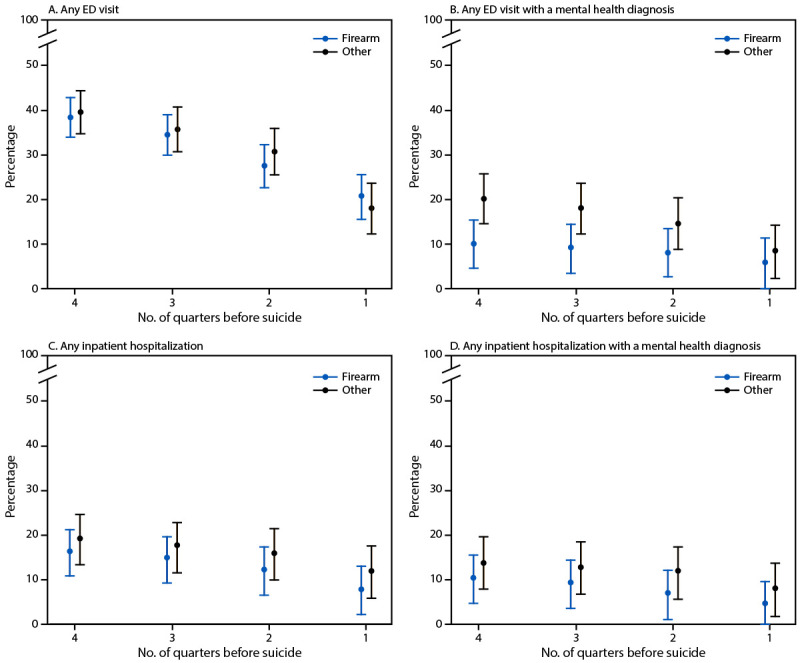
Percentage* of suicide decedents who had any emergency department visit (A), any emergency department visit with a mental health diagnosis (B), any inpatient hospitalization (C), or any inpatient hospitalization with a mental health diagnosis (D), in the year preceding suicide, by means of suicide (firearm or other) — three health care systems,^†^ Alaska, Colorado, and Washington, 2020–2022 **Abbreviation:** ED = emergency department. * Values are cumulative over the entire period; 95% CIs are indicated by bars. ^†^ Southcentral Foundation (Alaska) and Kaiser Permanente (Colorado and Washington).

Mental health care use patterns were broadly similar across study sites. Most suicide decedents had one or more outpatient visits preceding recorded in the EHR in the year preceding suicide, with comparable proportions among those who died by firearm (86.0%) and by other means (91.6%). Cumulative care-use plots, which showed patterns of all health care use in the year preceding suicide death, including primary care, were similar for persons who died by firearm and other suicide means, including any primary care; however, fewer persons who died by firearm suicide than persons who died by other suicide means received any mental health care in specialty or primary care settings (Figure 1). In the four quarters before suicide death, 18.2% versus 33.9% received mental health specialty care, and 26.3% versus 37.7% received primary care with a mental health diagnosis, respectively. Proportions of persons receiving any emergency or inpatient care were similar between groups (Figure 2). Across service settings, cumulative rates of past-year health care use among persons who died by suicide were highest for primary care (62.5%) (Supplementary Table 1), followed by emergency care (38.4%), outpatient mental health specialty care (24.6%), inpatient care (18.2%), and urgent care (16.5%). However, among persons who used care in these settings, the mean number of visits was highest among those who received mental health specialty care and was similar among those who died by firearm and those who died by other means of suicide (7.3 versus 10.9 visits; p = 0.10). The second highest mean number of visits was among those who received primary care and was also similar among those who died by firearm and other means (4.0 versus 4.4; p = 0.34). This was followed by the number of emergency care visits, which was lower among those who died by firearm (1.9 visits) than by other suicide means (2.9 visits; p<0.01). Sex-stratified analyses indicated that prevalences of primary care and outpatient mental health specialty care use were higher overall for females than for males (Supplementary Table 2). The mean number of health care visits by females and males were generally similar among those who died by firearm and those who died by other means. However, among females, those who died by firearm tended to have fewer mental health care specialty visits (mean = 5.8–12.2 during the 3, 6, and 12 months preceding death) than did females who died by other suicide means (mean = 6.2–16.3).

## Discussion

Among suicide decedents who received care through three health care organizations in Alaska, Colorado, and Washington, health care use patterns in the year preceding death were similar among those who died by firearm and those who died by other means, with the exception of mental health care use, which was significantly lower among persons who died by firearm suicide than among those who died by other suicide means. Moreover, less than one fourth (24.6%) of all persons who died by suicide received mental health specialty care, highlighting the importance of identifying opportunities for suicide prevention during other types of health care encounters, such as primary and emergency care encounters.

This study builds on previous research studies that found high rates of primary care use in the year preceding suicide death (*1*), as well as lower likelihood of having diagnosed mental health conditions, substance use conditions, and suicide attempts documented in medical records among persons who die by firearm suicide compared with those who die by other suicide means (*6*,*7*). Another recent study demonstrated that implementation of depression screening followed by suicide risk assessment and safety planning in primary care settings resulted in a 25% reduction in the suicide attempt rate in the 90 days following primary care visits (*8*,*9*). However, evidence for the effectiveness of these practices in longer-term suicide prevention (i.e., in the year after health care encounters) is limited. The findings in this report highlight the possible value of primary care–based suicide prevention practices for firearm suicide prevention specifically, because those who died by firearm suicide were less likely to have received care in outpatient specialty mental health care settings than were those who died by other suicide means.

Suicide risk screening and assessment, when combined with safety planning, appear helpful for prevention (*9*). However, some patients only experience suicidal thoughts a short time before their suicide attempt (*10*), and risk identification practices, such as screening for suicidal thoughts, might miss some patients at risk for suicide but who are not experiencing suicidal thoughts at the time of their health care encounters, particularly when these encounters do not occur in close proximity to events precipitating suicide attempts. Future research exploring how to improve identification and engagement of patients at risk for suicide across care settings, specifically in primary and emergency care settings, will be needed for continued practice improvement.

### Limitations

The findings in this report are subject to at least two limitations. First, this analysis included three health care systems serving populations with demographic characteristics (i.e., sex, age, insurance status, and medical conditions) that might not be generalizable to other U.S. health care systems. Second, the study period included the year 2020, when the COVID-19 pandemic temporarily affected trends in health care use. 

### Implications for Public Health Practice

This report highlights how suicide prevention practices in health care, designed to help identify and engage persons at risk in supportive care, need to reach beyond mental health encounters, particularly for firearm suicide prevention. These findings are guiding the development and implementation of suicide prevention practices in the systems included in this analysis and could be similarly useful to other health care systems in efforts to reduce the second leading cause of death among persons aged 10–44 years in the United States. For persons in crisis, help is available through the Substance Abuse and Mental Health Services Administration’s 988 Suicide and Crisis Lifeline or by texting or calling 988).

## Footnotes

* Web-based Injury Statistics Query and Reporting System Leading Causes of Death – CDC; Web-based Injury Statistics Query and Reporting System Leading Causes of Death in the U.S., 1981–2023 – CDC

†
Customer-Ownership – Southcentral Foundation

§
ICD-10 Classification – World Health Organization

¶
2024 Diagnosis Codes – Mental Health Research Network
